# Effect of the Addition Frequency of 5-Azacytidine in Both Micro- and Macroscale Cultures

**DOI:** 10.1007/s12195-020-00654-9

**Published:** 2020-10-06

**Authors:** Sandeep Kadekar, Laurent Barbe, Martin Stoddart, Oommen P. Varghese, Maria Tenje, Gemma Mestres

**Affiliations:** 1 Department of Chemistry-Ångström Laboratory, Uppsala University, 751 21 Uppsala, Sweden; 2 Department of Materials Science and Engineering, Science for Life Laboratory, Uppsala University, Box 35, 751 03 Uppsala, Sweden; 3 AO Research Institute Davos, 7270 Davos, Switzerland

**Keywords:** Differentiation, Mesenchymal stem cells, Microfluidics, Multilineage, Priming

## Abstract

**Introduction:**

Human mesenchymal stem cells (hMSCs) have a great clinical potential for tissue regeneration purposes due to its multilineage capability. Previous studies have reported that a single addition of 5-azacytidine (5-AzaC) causes the differentiation of hMSCs towards a myocardial lineage. The aim of this work was to evaluate the effect of 5-AzaC addition frequency on hMSCs priming (i.e., indicating an early genetic differentiation) using two culture environments.

**Methods:**

hMSCs were supplemented with 5-AzaC while cultured in well plates and in microfluidic chips. The impact of 5-AzaC concentration (10 and 20 *μ*M) and addition frequency (once, daily or continuously), as well as of culture period (2 or 5 days) on the genetic upregulation of PPARγ (adipocytes), PAX3 (myoblasts), SOX9 (chondrocytes) and RUNX2 (osteoblasts) was evaluated.

**Results:**

Daily delivering 5-AzaC caused a higher upregulation of PPARγ, SOX9 and RUNX2 in comparison to a single dose delivery, both under static well plates and dynamic microfluidic cultures. A particularly high gene expression of PPARγ (tenfold-change) could indicate priming of hMSCs towards adipocytes.

**Conclusions:**

Both macro- and microscale cultures provided results with similar trends, where addition frequency of 5-AzaC was a crucial factor to upregulate several genes. Microfluidics technology was proven to be a suitable platform for the continuous delivery of a drug and could be used for screening purposes in tissue engineering research.

**Electronic supplementary material:**

The online version of this article (10.1007/s12195-020-00654-9) contains supplementary material, which is available to authorized users.

## Introduction

The multilineage capacity of mesenchymal stem/stromal cells (MSCs), first suggested by Cohnheim in 1867, resulted in a new paradigm in the field of tissue engineering with high clinical potential.^38^ Nowadays there are consolidated *in vitro* protocols that enable the differentiation of hMSCs into mesodermal cell types such as osteoblasts, chondrocytes, adipocytes and myoblasts.^5^,^6^ In the recent years, the approach of priming hMSCs (also referred as preconditioning or licensing), meaning to prepare the cells for some lineage-specific differentiation, have started to be investigated. Cell priming can be achieved using pharmacological drugs and it has the potential to increase MSCs therapeutic efficiency.^22^

Priming and even differentiation of hMSCs *in vitro* are influenced by several parameters such as the active compound used, its concentration and frequency of addition, as well as the culture period. While countless compounds and the effect of their concentration have been reported, the addition frequency has not been thoroughly studied. The standard methodology consists of manually adding a discrete dose in a cell culture vessel. One of the main drawbacks of this procedure is that the concentration of the active compound decreases over time, effect linked to its stability and the interaction with cells.

To maintain a constant concentration of an active compound over time, bioreactors or microfluidic systems, which are able to provide a dynamic media, can be employed. Another advantage of dynamic platforms is that the *in vivo* conditions can be partially mimicked^2^ by perfusing media through two-dimensional or three-dimensional scaffolds, which could potentially protect the original phenotype of hMSCs.^3^,^26^ In fact, bioreactors have been commonly explored in the field of tissue engineering, allowing for the production of living tissues that later can be implanted in patients.^17^ Compared to bioreactors, the smaller size of microfluidic systems permits minimizing cell numbers and reagents. The low number of cells needed in microfabricated systems is especially relevant when working with primary hMSCs, since the significantly shorter required expansion minimizes the variabilities of the phenotype.^24^ MSCs have previously been differentiated in microscale cultures using simultaneous chemical and mechanical (i.e., shear stress) stimuli, towards chondrogenic,^4^ adipogenic^21^,^40^ and neuronal lineage.^35^

5-Azacytidine (5-AzaC) was synthesized in 1964 as nucleoside antimetabolite with clinical specificity for acute myelogenous leukemia.^29^ Upon uptake by cells, 5-AzaC is incorporated into the DNA and inhibits DNA methylation.^7^ The safety profile of 5-AzaC was proven and in 2004 it was approved by the U.S. Food and Drug Administration for the treatment of all subtypes of myelodysplastic syndrome (MDS), a group of cancer caused by epigenetic changes in DNA structure.^11^ In addition, 5-AzaC has previously shown its potential to directionally differentiate MSCs towards myocytes or cardiomyocytes *in vitro.*^16^,^18^,^20^,^25^,^34^,^39^ Differently, when culturing C3H10T1/2 (i.e., a MSCs cell line) with 5-AzaC, cells committed into three different lineages: adipogenic, myogenic and chrondrogenic.^33^

The aim of this work was to elucidate the effect of the addition frequency of 5-AzaC on the priming of hMSCs (towards adipocyte, chondrocyte, myoblast or osteoblast lineage) when two culture environments were used, specifically static well plates and dynamic microfluidic cultures.

## Materials and Methods

### 5-Azacytidine

A stock solution of 500 *μ*M of 5-Azacytidine (5-AzaC; Sigma-Aldrich, A2385) was prepared by dissolving it in a non-supplemented Dulbecco’s Modified Eagle Medium (DMEM) with low content of glucose (Thermo Fisher Scientific, Gibco, ref. no. 10567014). The stock solution was used directly after preparation.

The capacity of 5-AzaC to prime hMSCs was evaluated at two different concentrations. 10 *μ*M was selected since it has been used to differentiate MSCs in several reported literature.^14^,^15^,^18^,^20^,^25^,^34^,^42^,^43^ In addition, to determine the dose effect, a twofold concentration (20 *μ*M) was also employed.

### Fabrication of the Microfluidic Chip

**Figure 1:**
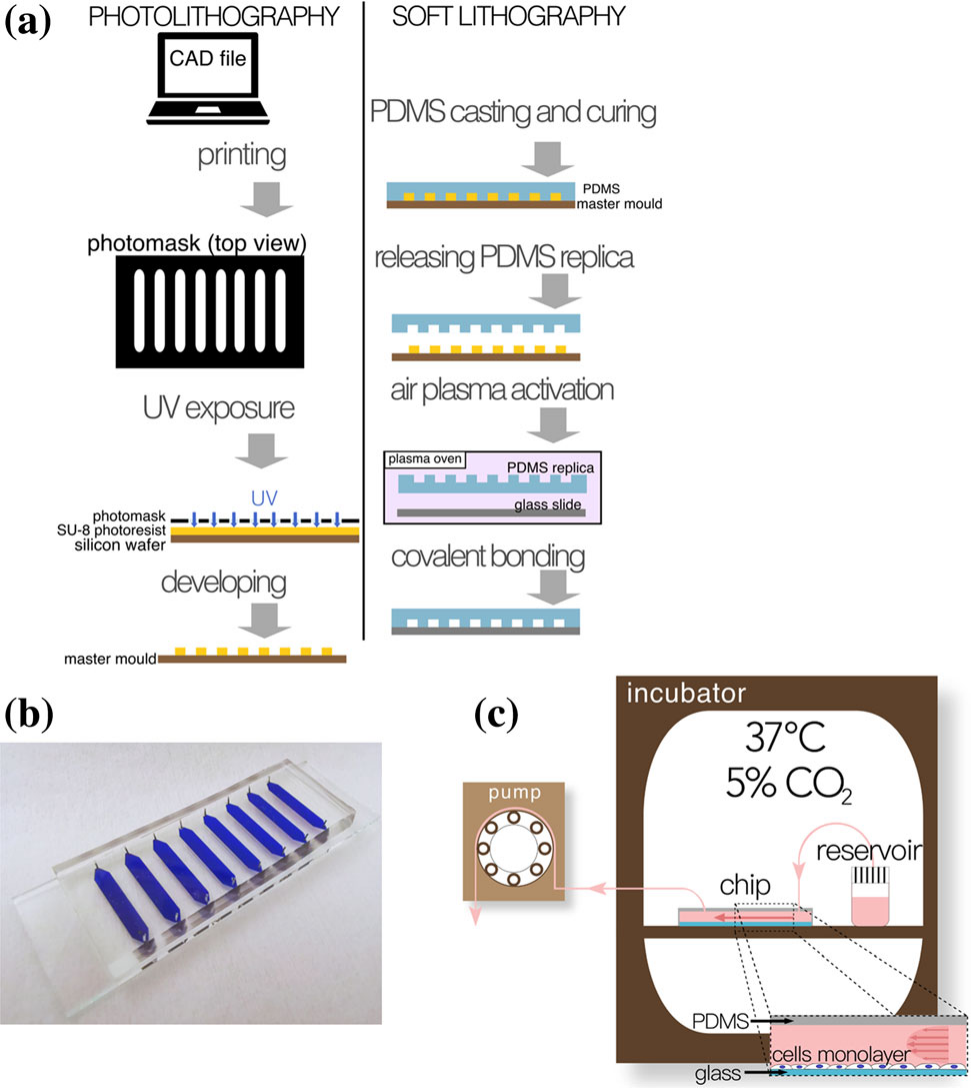
(**a**) Scheme of photolithography and soft lithography; (**b**) microfluidic chip; (**c**) microfluidic setup.

A microfluidic chip made of polydimethylsiloxane (PDMS) was fabricated using standard soft-lithographic techniques, and the PDMS channel structures were bonded onto a glass slide. Soft lithography using a SU-8 master on a silicon wafer was used for moulding the PDMS substrate. In short, the SU-8 master was prepared by depositing a SU-8 layer on the silicon wafer by a spinning process. Afterwards, the SU-8 was soft baked in two steps, at 65 and 95 °C. The non-exposed SU-8 pattern was developed using Mr-600 (Micro Resist Technology, ref. no. R815100) and a hard bake process was performed at 150 °C (Fig. 1a).

PDMS is a silicone material widely used to build microfluidic systems for cell culture since it is biocompatible, transparent and gas permeable, therefore allowing for the necessary oxygen exchange for cell culturing.^36^ PDMS (Dow Corning Sylgard^®^ 184) polymer was mixed with its curing agent in a 10:1 weight ratio and poured on the prepared SU-8 master. The PDMS polymer was cured at 60 °C for 2 h. Inlets and outlets of 0.5 mm diameter were prepared using a biopsy punch (World Precision Instruments, ref. no. 504528). Finally, both the PDMS slab and the glass slide were treated in a plasma oven for 1 min to activate their surfaces and they were covalently bonded together thus forming a sealed microfluidic chip. The bonding was strengthened by heating the assembled chip overnight at 100 °C (Fig. 1a).

The chip design comprised of eight independent channels with the following dimensions: length of 20 mm, width of 3 mm (constant width along 15 mm) and height of 200 *μ*m (Fig. 1b, Table 1). The width in the inlet and outlet gradually increased from 0.5 to 3 mm to avoid a dramatic change in geometry that could cause dead volumes.

**Table 1:** Differences between a macroscale culture (well plate) and a microscale culture (microfluidic chip) regarding substrate material, dimensions (surface, volume, surface-to-volume ratio), cell density, flow applied and its resulting shear stress

Macroscale and microscale culture	Substrate material	Surface (mm^2^)	Volume of media (*μ*L)	Surface-to-volume ratio (*μ*L/mm^2^)	Cell density (cells/cm^2^)	Flow applied (*μ*L/min)	Shear stress (dyn/cm^2^)
12-well plate (well)	Culture-treated polystyrene	380	1000	2.63	30,000	N/A	N/A
Microfluidic chip (channel)	Gelatin-coated glass (channel structures created in PDMS)	52.5	10.5	0.2	30,000	1	0.007

The 8 channels of the chip were connected to a peristaltic pump (Shenchen, LABV1/MC12(10)), as depicted in Fig. 1c. The actual setup is shown in Fig. SM 1. The media (either 5-AzaC-loaded medium or plain medium) was pulled from individual small reservoirs. The pump ran continuously along the entire experiment, maintaining a constant flow of 1 *μ*L/min, and being only stopped for a short period of time once a day to refresh the media in the reservoirs.

Wall shear stress applied to the cells is calculated assuming Newtonian fluid and parallel-plate geometry, using a simplified equation (Eq. 1)^9^:

1 $$\tau_{\text{WSS}} = \frac{6Q\mu }{{wh^{2} }},$$

where *τ* is the wall shear stress (dynes/cm^2^), *Q* is the flow rate (m^3^/s), *μ* is the viscosity of a DMEM culture media at 37 °C (8.4 × 10^−4^ Pa s^9^), *w* is the channel width (m) and *h* is the channel height (m). In the microfluidic chip designed, a flow of 1 *μ*L/min resulted in a wall shear stress of approximately 0.007 dyn/cm^2^.

### Cell Culture

Human bone marrow-derived mesenchymal stem cells (hMSCs), obtained from five different donors, were mixed and cultured together as described earlier.^8^ hMSCs were isolated from bone marrow of healthy donors after full ethical approval (ethical approval KEK ZH-NR: 2010-0444/0). DMEM low glucose medium (ThermoFisher, Gibco ref. no. 10567014) supplemented with 10% MSCs-qualified fetal bovine serum (ThermoFisher, Gibco ref. no. 12662029) and 1% penicillin/streptomycin (ThermoFisher, Gibco ref. no. 15140122) was used as culture medium (from now onwards named plain medium). Cells were maintained in cell culture flasks in an incubator with a humidified atmosphere of 5% CO_2_ in air at 37 °C. Plain culture medium was refreshed every second day and upon 80% confluency cells were detached with a minimum amount of TrypLE for 2–3 min. All experiments were done with cell passage between 2 and 4.

### Experiments in Well Plate

**Figure 2:**
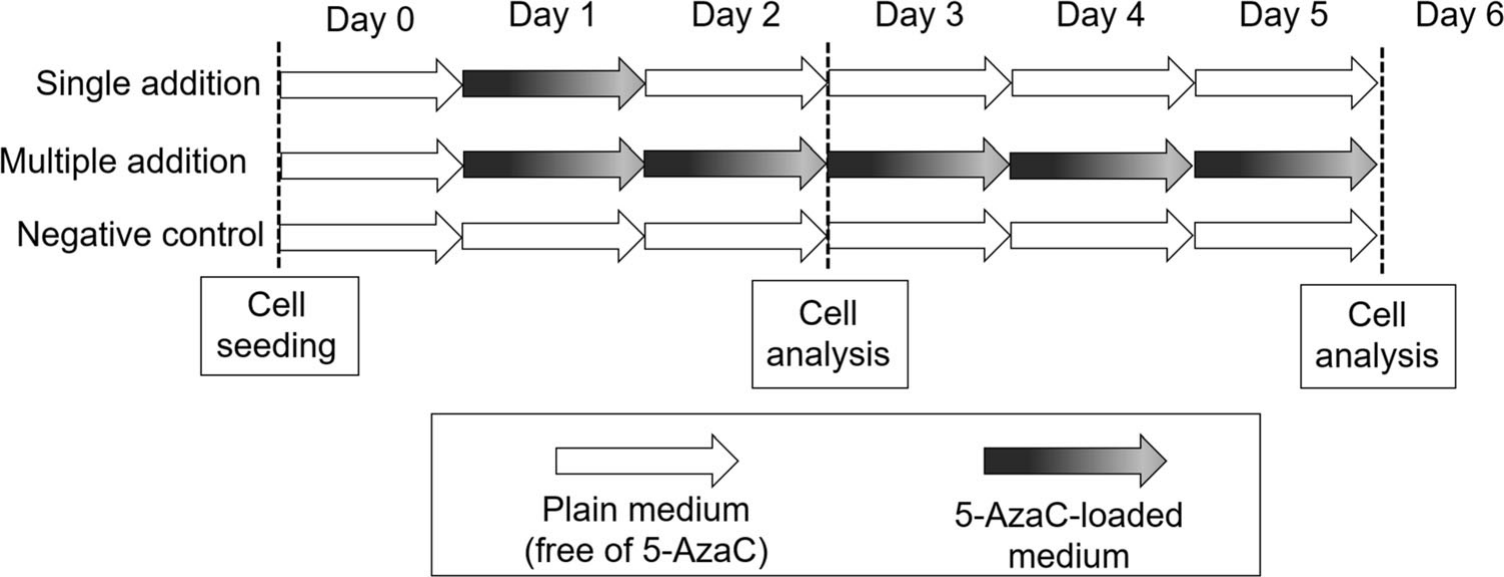
Experimental design showing the addition of 5-AzaC to the cell culture medium. In all conditions, media was daily replaced. While in the well plate 5-AzaC was added directly into the wells after media replacement, the entire 5-AzaC-loaded medium of the containers was replaced daily when using a microfluidic chip.

100,000 cells were seeded in 12-well tissue-culture treated plates (~ 30,000 cells/cm^2^) in 1 mL of plain culture media (Table 1). Twenty-four hours after cell seeding, cells were supplemented with an appropriate volume of the stock solution of 5-AzaC (500 *μ*M) to provide a drug concentration of either 10 or 20 *μ*M. To determine the importance of refreshing the active compound along a treatment, either media containing 5-AzaC (multiple additions of 5-AzaC) or plain media (a single addition of 5-AzaC) was daily replaced. Negative control samples for the qPCR calculations consisted of cells cultured in plain media (free of 5-AzaC) during the entire culture (Fig. 2).

The cytotoxicity of 5-AzaC was evaluated with PrestoBlue^®^ Cell Viability Reagent. In parallel, using some other well plates, total RNA was isolated from cells by adding 350 *μ*L of lysis buffer (QIAGEN, Germany) into the wells, directly followed by homogenization of cell lysates.

Duplicates were included in each experiment and the whole experiment was independently performed twice.

### Experiments in a Microfluidic Chip (On-Chip)

The channels of the microfluidic chip were coated with type A gelatin (Sigma G9136). Briefly, 0.2 wt% type A gelatin was dissolved in distilled water and autoclaved. 1 mL of gelatin solution was pipetted into the channels, incubated at 37 °C for 30 min and afterwards rinsed with PBS. 30 *μ*L of a highly concentrated cell suspension (1.5 × 10^6^ cells/mL) was carefully pipetted into each channel resulting in a cell suspension of 30,000 cells/cm^2^ (Table 1). 5-AzaC-loaded medium was added to all channels 24 h after cell seeding. After 1 day, half of the channels continued with 5-AzaC-loaded medium, which was daily replaced (multiple additions), and the medium in the other half of the channels was exchanged for plain medium (a single addition of 5-AzaC in total). Negative control samples for the qPCR calculations consisted of cells seeded on-chip and cultured in plain medium (free of 5-AzaC) along the entire period (Fig. 2). The media replacement was done either in a discrete and manual manner (with a pipette in a daily basis) or continuously at a flow rate of 1 *μ*L/min (media pulled from individual reservoirs, which were kept in the incubator and the containing media was daily replaced). The chips in static conditions, which media was manually replaced, were placed in a Petri dish containing a wet tissue to prevent media evaporation.

The experiment was stopped 5 days after addition of 5-AzaC. After disconnecting the chips, total RNA was isolated from cells by adding 30 *μ*L of lysis buffer (QIAGEN, Germany) per channel, directly followed by homogenization of cell lysates, and extraction of the solution through the outlet.

Triplicates were included in each experiment and the whole experiment was independently performed three times.

### Real-Time qPCR

Real-time qPCR enables amplification and quantification of a specific nucleic acid sequence. To evaluate the priming of MSCs towards adipocytes, chondrocytes, myoblasts and osteoblasts the master genes selected were PPARγ, SOX9, PAX3 and RUNX2, respectively. The house-keeping gene (ACTB) was used as an internal control. All genes used were TaqMan primers (Thermo Fisher Scientific, Sweden).

RNA was extracted from cell lysates using RNeasy Mini Kit (QIAGEN, Germany). RNA concentrations were determined by means of NanoDrop 2000 (Thermo Fisher Scientific, Sweden), with resulting optical density (OD) 260/280 ratios around 1.8.

Between 30 and 100 ng of the total RNA was used to make cDNA. The cDNA was prepared using a high-capacity RNA-to-cDNA kit following the manufacturer’s protocol (Applied Biosystems, USA). Real-time qPCR was performed with cDNA and TaqMan Fast Universal PCR Master Mix (2×) (Applied Biosystems, USA). The real-time PCR reactions were carried out with 10 *μ*L of 2 × TaqMan Universal PCR Master Mix, AmpErase uracil-*N*-glycosylase (UNG) (Applied Biosystems, USA), 5 *μ*L diluted cDNA, and 1 *μ*L of TaqMan gene-specific assay mix (Applied Biosystems, USA) in a 20 *μ*L final reaction volume. Amplification was carried out using the CFX Connect System (Bio-Rad, Sweden) using a 40-cycle program.

The CFX Manager software automatically calculates the raw C_t_ values. Only data from samples with a *C*_t_ value equal to or below 35 were further analyzed. Differences in cycle number thresholds were calculated using the comparative quantitation 2 − ΔΔ*C*_t_ method. Gene upregulation was measured in terms of fold change by calculating first Δ*C*_t_ (Eq. 2), followed by ΔΔ*C*_t_ (Eq. 3) and, finally, the fold change (Eq. 4). The gene was considered to be upregulated if the fold change was higher than 1.5.

2 $$\Delta C_{\text{t}} = C_{\text{t}} \left( {\text{target}} \right){-}C_{\text{t}} \left( {{\text{endogenous}}\;{\text{control}}} \right),$$

3 $$\Delta \Delta C_{\text{t}} = \Delta C_{\text{t}} \left( {\text{target}} \right){-}\Delta C_{\text{t}} \left( {\text{NC}} \right),$$

4 $${\text{Fold}}\;{\text{change}} = 2 - \Delta \Delta C_{\text{t}} ,$$

where *C*_t_ (target) and *C*_t_ (endogenous control) are the cycle threshold for the target gene and for ACTB, respectively, and NC is the negative control. All samples were performed in duplicate.

### Statistics

Statistical analyses were performed in Minitab 18 (Statistical Software). One-way ANOVA method at a significance level of *α* = 0.05, followed by Tukey’s *post hoc* test was performed to compare the statistically significant differences among the groups. Data are reported as mean ± standard deviation.

## Results

### Cytotoxicity of 5-AzaC

**Figure 3:**
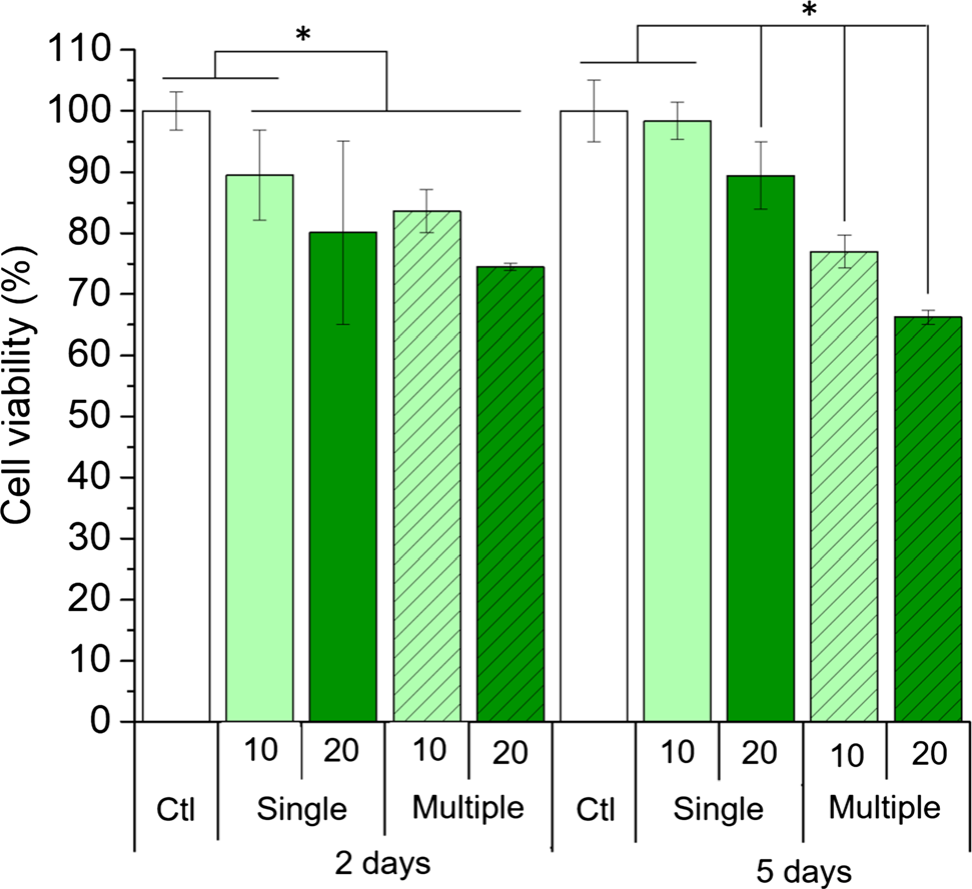
Viability of hMSCs exposed to 10 and 20 *μ*M 5-AzaC, which was added in a single or multiple occasions over a culture period of 2 and 5 days. The data was normalized to the control, where cells were cultured with plain medium. Ctl stands for control. *indicates significant differences between a sample and the control within each time point (*p* < 0.05).

The cytotoxicity of 5-AzaC was dependent on its concentration and frequency of addition. A single addition of 10 or 20 *μ*M 5-AzaC caused a decrease of maximum a 20% of the cells at 2 and 5 days. The highest cell death was observed with multiple additions of 20 *μ*M 5-AzaC, which killed 25% or 33% of the cells after 2 or 5 days, respectively (Fig. 3 and *p*-values in Table SM 1). These results are in good correlation with the lower number of cells observed by bright field imaging after multiple additions of 5-AzaC (Fig. SM 2) and are in line with previous studies.^10^,^25^,^39^,^42^

### Priming hMSCs with 5-AzaC in Well Plates

**Figure 4:**
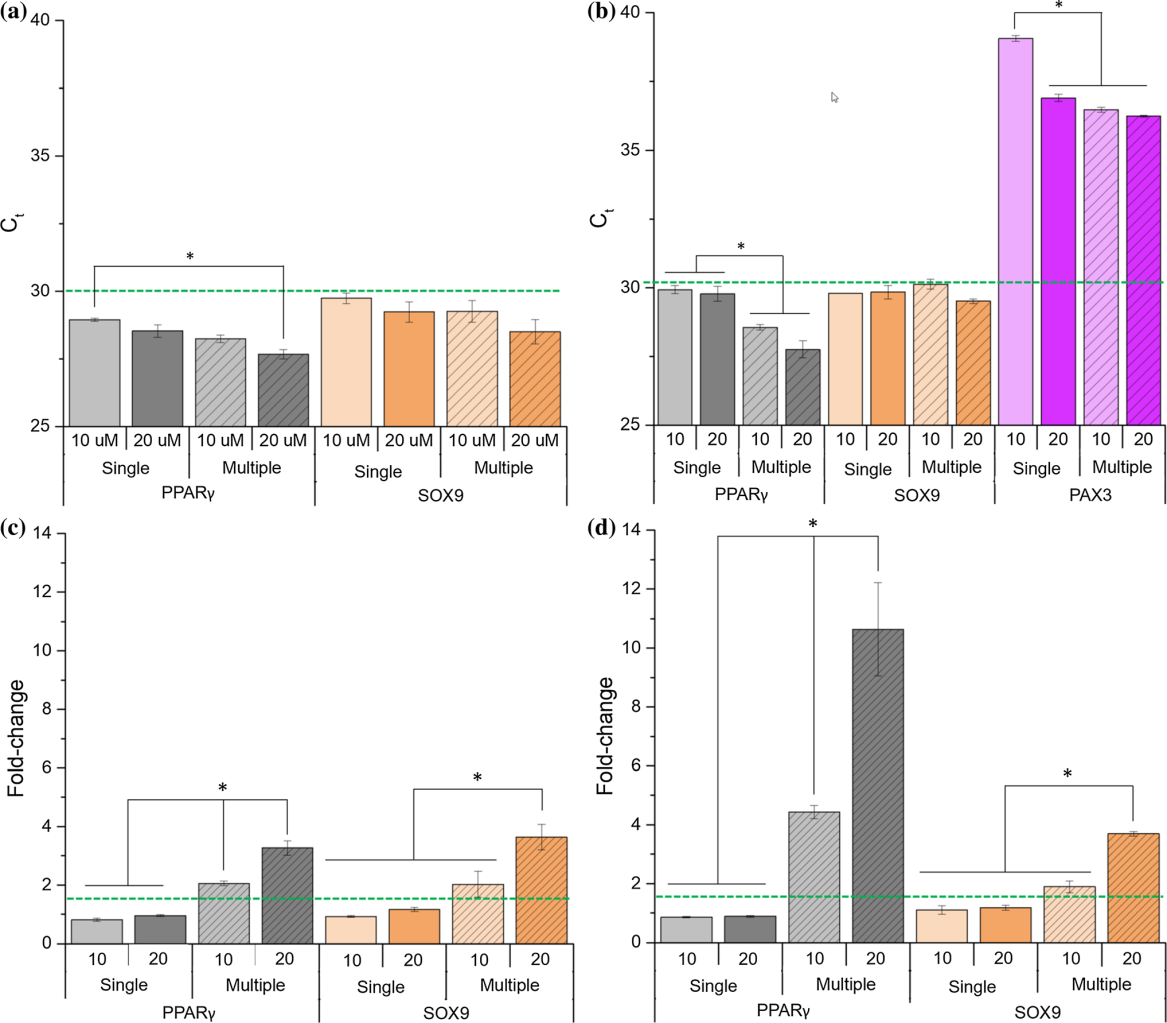
qPCR assessment of PPARγ, SOX9 and PAX3 genes for hMSCs cultured with 5-AzaC at different concentrations (10 and 20 *μ*M), which was supplemented only once (single addition) or daily (multiple additions) for 2 or 5 days in a 12 well plate. Data represented as cycle threshold (*C*_t_) for cultures in which 5-Aza was supplemented for (**a**) 2 days, (**b**) 5 days, and as fold change for cultures containing 5-AzaC for (**c**) 2 days, (**d**) 5 days. The upregulation level (fold change > 1.5) is marked with a dash line. * indicates statistically significant differences (*p* < 0.05) within each master gene.

The effect of 5-AzaC in the priming of hMSCs was evaluated 2 days and 5 days after a single or multiple addition of 5-AzaC. The cycle threshold (*C*_t_) for both PPARγ and SOX9 was under 30 at both time points (Fig. 4a). In contrast, the *C*_t_ values of PAX3 were between 36.2 and 39.1 after 5 days (Fig. 4b), representing a low upregulation of this gene. For this reason, fold change data of PAX3 is not shown and this gene was excluded from further studies.

After 2 days, a single addition of 5-AzaC (10 or 20 *μ*M) did not upregulate PPARγ or SOX9 (fold change < 1.5). In contrast, multiple additions of 5-AzaC resulted in upregulation (fold change > 1.5) of both PPARγ and SOX9. Specifically, multiple additions lead to statistically higher upregulation than a single addition at 10 *μ*M (*p* = 0.002 for PPARγ) and 20 *μ*M (*p* < 0.001 for PPARγ and *p* = 0.005 for SOX9), with the higher concentration almost doubling the fold change. The upregulation level was similar for PPARγ and SOX9 comparing analogous concentrations and addition frequency of 5-AzaC (Fig. 4c). Similarly, after 5 days of 5-AzaC addition, no upregulation was observed (fold change < 1.5) when a single addition was done, independently of the concentration or master gene. In contrast, multiple additions of 20 *μ*M 5-AzaC led to significant upregulation of both PPARγ and SOX9 genes in comparison to a single addition (*p* = 0.013 for PPARγ; *p* = 0.002 for SOX9). The higher concentration of 5-AzaC (20 *μ*M) strengthened the priming effect in comparison to the lower concentration (10 *μ*M) when multiple additions were performed (*p* = 0.001 for PPARγ; *p* = 0.003 for SOX9) (Fig. 4d).

### Priming hMSCs with 5-AzaC in Microfluidic Chips

Control experiments were performed to rule out the effect of channel coating with gelatin (Fig. SM 3) and shear stress (Fig. SM 4). After confirming that the flow shear stress applied to hMSCs did not cause upregulation of the genes of interest, cells were cultured on-chip under discrete (daily, manually) and continuous (flow at 1 μL/min) delivering 5-AzaC-loaded medium for 5 days. The C_t_ values for the three genes (PPARγ, SOX9 and RUNX2) and the four conditions (discrete/continuous delivery; single/multiple addition) tested were between 28.5 and 32.3.

**Figure 5:**
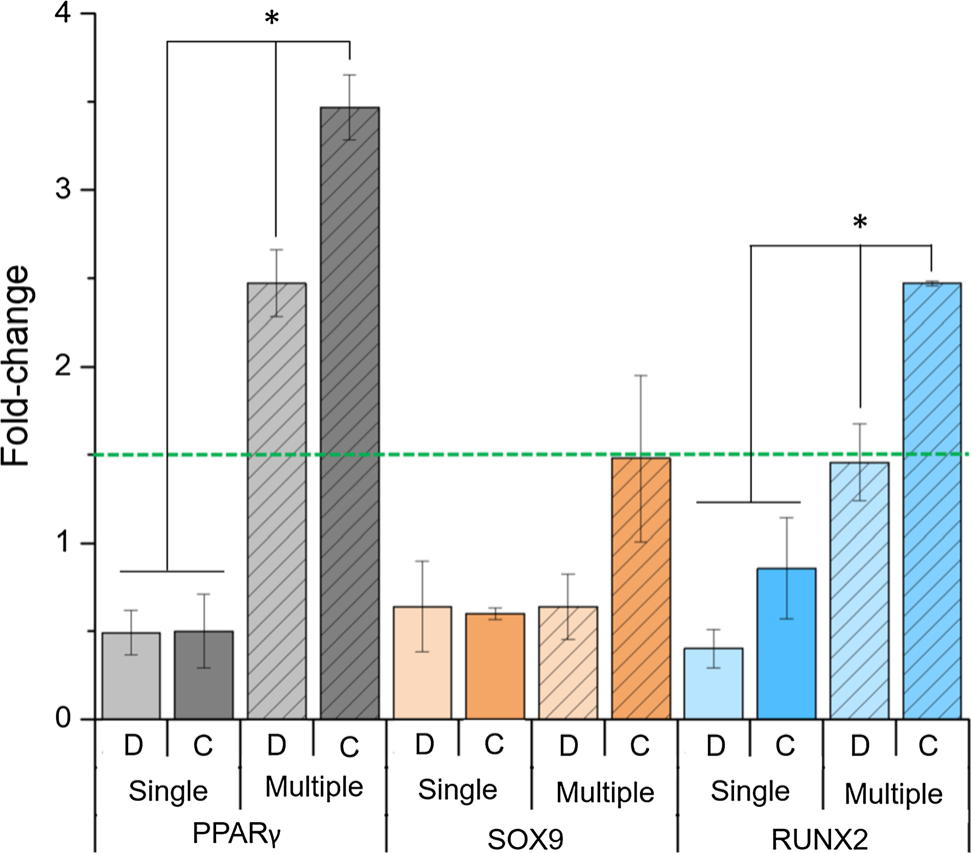
qPCR assessment of PPARγ, SOX9 and RUNX2 genes for hMSCs cultured on-chip with 5-AzaC (20 *μ*M) for 5 days, with discrete (D; daily, manual, static conditions) or continuous delivery (C; flow maintained at 1 *μ*L/min). 5-AzaC was added in the media either once (single addition) or in a daily basis (multiple additions). The upregulation level (fold change > 1.5) is marked with a dash line. * indicates statistically significant differences (*p* < 0.05) within each master gene.

Figure 5 shows that in case of a single addition, a similar gene expression was observed regardless of the delivery modality (discrete or continuous). Multiple additions of 5-AzaC caused a statistically significant increase of PPARγ (*p* = 0.004 if discrete, *p* < 0.001 if continuous) and RUNX2 (*p* = 0.034 if discrete, *p* = 0.004 if continuous) in comparison of making a single addition. When 5-AzaC was added in the reservoirs in multiple occasions, the continuous perfusion of 5-AzaC caused a substantial upregulation of the three genes in comparison with a discrete dispensing, namely 40.3% for PPARγ, 131.6% for SOX9 and 69.7% for RUNX2, and was statistically significant for both PPARγ (*p* = 0.046) and RUNX2 (*p* = 0.027).

## Discussion

5-AzaC is considered as an epigenetic drug since it has been found effective to promote the differentiation of MSCs towards myocytes and cardiocytes.^16^,^18^,^20^,^25^,^34^,^39^ In the majority of the previous *in vitro* studies, 5-AzaC has been supplemented to the cells on a single addition basis, and the effect on the addition frequency has not been studied. In this work the effect of addition frequency is explored using both static well plates and dynamic microfluidic systems.

The cytotoxicity of 5-AzaC reported by other studies^10^,^25^,^39^,^42^ was also confirmed in this work (Fig. 3). Therefore, the number of additions should be optimized to achieve priming towards the cell lineage of interest while minimally compromising cytotoxicity.

Looking at the genetic upregulation of the well plate cultures, similar levels were observed for both PPARγ and SOX9 2 days after 5-AzaC addition when analogous concentrations and addition frequencies were used. This resemblance in upregulation was associated to the short-term of the culture.^13^ Therefore, upregulation was evaluated at a later time point of 5 days (Fig. 4d). Cultures displayed an upregulation higher than tenfold-change for PPARγ when daily additions of 5-AzaC 20 *μ*M were performed over 5 days (Fig. 4d). Such a high upregulation suggested that priming (phenomenon that occurs before gene expression and lineage specification^37^) of hMSCs towards adipocytes occurred. This apparent contradiction between the current work and previous studies showing that 5-AzaC enhanced hMSCs differentiation towards myocytes or cardiomyocytes could be mainly ascribed to three parameters: the addition frequency (usually a single addition), concentration (often 3 or 10 *μ*M) and culture period (in general between 1 and 28 days).^16^,^20^,^25^,^34^

Multiple additions of 5-AzaC promoted a statistically significant increase in gene expression for all genes tested in comparison to a single addition, this applying to both concentrations (10 and 20 *μ*M) of 5-AzaC, culture types (well plate and on-chip), delivery frequency on-chip (discrete and continuous) and time points (2 days and 5 days) (Figs. 4c, 4d, and 5). Specifically, for PPARγ, the effect of making daily additions of 5-AzaC (20 *μ*M) in the well plate in comparison to a single addition caused a fold change increase of 240.7% at day 2 and 1103.6% at day 5. The relevance of multiple additions can be explained by the short half-life (*t*_1/2_) of 5-AzaC, which has been reported to be of 7 h at 37 °C *in vitro.*^32^ Therefore, theoretically, daily additions of 20 *μ*M of 5-AzaC for 5 days would guarantee that a concentration ≥ 10 *μ*M would be maintained over ~ 30% of the culture period, which was shown to be enough to promote upregulation. In contrast, a single addition over 5 days would theoretically lead to a concentration ≥ 10 *μ*M over only ~ 6% of the culture period, which did not seem successful to enhance upregulation.

The drastic increase in gene upregulation when multiple additions of 5-AzaC were performed over time suggested that a continuous addition of the drug could lead to an even higher upregulation. For this purpose, a microfluidic chip that allowed a continuous delivery of 5-AzaC was designed. It is noteworthy to highlight that the container with 5-AzaC-loaded media connected to the microfluidic chip was maintained in the incubator, meaning that the degradation rate of the drug would be the same on-chip than in the well plate. Herein, a similar effect of 5-AzaC was expected on both culture formats. In the future, by upgrading the design of the chip, the drug could be added in powder and dissolved in-line right before getting in contact with the cells.

Microfluidic systems have several intrinsic advantages for cell culturing, including the low amount of cells needed.^19^ This is especially important for primary MSCs, since it decreases the need of expansion of isolated MSCs, process that dramatically alters hMSCs properties and therefore reduces the prediction value of the true differentiation capacity of MSCs *in vitro.*^24^ Another characteristic of microfluidic chips is that the cultured cells may be exposed to shear stress resulting from the flow. The conditions in which hMSCs have been previously cultured in a microscale environment varies enormously between works, with regards to shear stress levels and culture period. Shear stress values between 0.002 and 22 dyn/cm^2^ have been previously applied to hMSCs, usually for a period of up to 13 days.^12^,^27^,^28^,^41^ In the current study, the aim of using a microfluidic chip was to evaluate whether a continuous delivery of 5-AzaC would increase the genetic upregulation in comparison to a manual addition of the drug. Herein, it was important to first discard any potential gene upregulation due to shear stress. For this reason, a low flow rate of 1 *μ*L/min was selected to induce a low shear stress of 0.007 dyn/cm^2^. In these conditions, the flow did not seem to upregulate any of the genes tested (fold change < 1.5) (Fig. SM 4), although RUNX2 showed the highest fold change of the three genes, in good correlation with literature.^12^,^27^,^28^,^41^

Culturing hMSCs on-chip with a continuous delivery of 5-AzaC (loaded-media changed daily) enhanced a higher upregulation of PPARγ, SOX9 and RUNX2 in comparison to culturing the cells on-chip in static conditions (media refreshed daily) (Fig. 5). The higher upregulation was ascribed to the full replenishment of 5-AzaC-loaded medium ~ 6 times per hour, minimizing the decrease of 5-AzaC concentration due to the cellular uptake. Interestingly, the lack of accumulation of signaling molecules and secreted factors due to a continuous flow^1^ did not seem to impede the genetic upregulation. To partially overcome the degradation of 5-AzaC in the reservoirs, the 5-AzaC-loaded medium was refreshed every 24 h. The daily change of the media was proven to be of great importance while culturing cells on-chip, leading to an increase of 593.6% for PPARγ, 146.5% for SOX9 and 188.8% for RUNX2. In fact, none of the genes were upregulated if 5-AzaC was only added once in the media. This was again associated to the short half-life time of 5-AzaC, as previously discussed.

The consequences of using dynamic microscale cultures in comparison to static standard well plates is not trivial. Stangegaard *et al.* investigated the phenotypic differences of cells cultured in microfluidic systems and compared it to the well plates. The results showed that human cervical carcinoma cells (HeLa) grown in a microscale culture environment were biologically comparable (almost identical gene expression profile) than cells grown in a culture flask.^30^ The same research group also concluded that using PDMS as a substrate did not either influence gene expression profiles of HeLa cells in comparison to cell culture flasks.^31^ Although these results cannot be generalized to all cell types, they provided reliability to the outcome of on-chip systems.

In our study, both the well plates (Fig. 4d) and the microfluidic chips (Fig. 5) resulted in similar trends regarding the upregulation of the master genes studied but differed significantly in absolute numbers. For example, performing multiple discrete additions of 5-AzaC for 5 days, the upregulation of PPARγ was four times higher in well plates than in on-chip cultures (both under static conditions). The difference in absolute numbers can be explained by the different environment of a micro- and a macroscale culture,^23^ namely the surface-to-volume ratio, shear stress, nutrient supply, waste removal, concentration of secreted factors, *etc*.^23^ Some of these parameters could potentially cause upregulation of several proteins sensitive to a wide variety of stressors, affecting the cells differently depending on their environment.^23^

The current study proves the importance of testing cell response to different addition frequencies of a drug, procedure that is often omitted. The genetic upregulation observed in this work, which was particularly high for PPARγ (tenfold increase observed) seemed to be indication that priming towards adipocyte lineage occurred. In future studies, a differentiation assay should be performed, which may require supplementing the culture media with differentiation factors and the characterization of the cells at longer time points. For example, adipocytes could be identified by Oil red O staining to observe the high levels of intracellular lipid accumulation as well as by immunostaining of proteins such as PPARγ. This work also proves that microfluidic systems can be used to evaluate hMSCs priming when continuously delivering a drug. Therefore, microfluidic systems could be used first to screen drugs or even cells isolated from patients. In a second phase, bioreactors could be employed to scale up the process and potentially engineer tissue using a cell-seeded scaffold approach.

## Conclusions

The relevance of performing repetitive additions of a drug to enhance the upregulation of a gene have been demonstrated. In particular, daily additions of 20 *μ*M 5-AzaC to hMSCs lead to a dramatic increase in PPARγ expression (~ tenfold increase after 5 days) as well as the upregulation of SOX9 and RUNX2, which contrasts with the lack of gene upregulation when 5-AzaC was added in a single dose. The gene upregulation was also strengthened by the higher 5-AzaC concentration, but only in the case of multiple additions. The ability of 5-AzaC to strongly upregulate PPARγ could indicate priming towards an adipogenic lineage, unraveling a new dimension of 5-AzaC for tissue regeneration applications. Additionally, a microfluidic system was used to provide a favorable microscale environment for cell culture and to continuously deliver 5-AzaC to hMSCs. The upregulation trends were the same in the microfluidic chips as in the well plates, although absolute numbers could not be directly compared due to the different culture conditions. Proving that microfluidic systems can be used for screening purposes may open a new application for this technology, becoming an important tool to understand dose-dependent effects of drugs on therapeutic cells.

## Electronic supplementary material

Below is the link to the electronic supplementary material.

Supplementary material 1 (pdf 387 kb)
