# Supplementary material for: Effect of the Addition Frequency of 5-Azacytidine in Both Micro- and Macroscale Cultures
Source: Cell Mol Bioeng. 2020 Oct 6;14(1):121–30. doi: 10.1007/s12195-020-00654-9 (PMC7878657; doi:10.1007/s12195-020-00654-9)
Supplement: Supplementary file 1 — Supplementary material 1 (pdf 387 kb) [file 12195_2020_654_MOESM1_ESM.pdf]

Effect of the addition frequency of 5-azacytidine in both micro- and macroscale cultures

Sandeep Kadekar[1], Laurent Barbe[2], Martin Stoddart[3], Oommen P. Varghese[1], Maria Tenje[2], Gemma Mestres[2*]

[1]Department of Chemistry-Ångström Laboratory, Uppsala University, 751 21, Uppsala, Sweden

[2] Department of Materials Science and Engineering, Science for Life Laboratory, Uppsala University, Box 35, 751 03 Uppsala, Sweden

[3]AO Research Institute Davos (ARI), AO Foundation, Clavadelerstrasse 8, 7270 Davos, Switzerland

* Gemma.mestres@angstrom.uu.se

Supplementary material (SM)

Materials and methods

Actual setup used to culture hMSCs with 5-AzaC-loaded media in microfluidic chips.

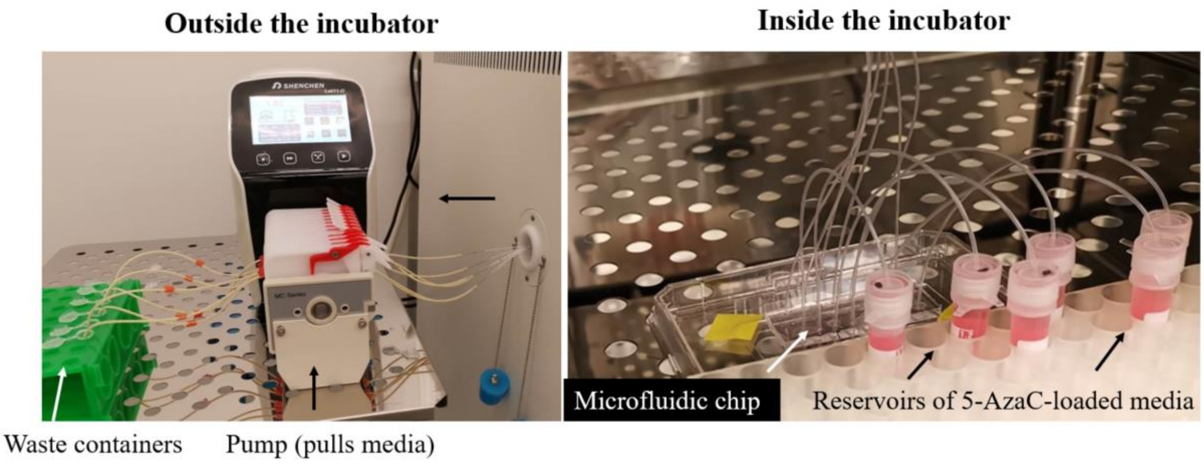

Figure SM 1. Picture of the setup outside and inside the incubator.

Results

Morphology of hMSCs cultured with 5-AzaC-loaded medium

The morphology of hMSCs cultured with 20 µM 5-AzaC was evaluated by bright field imaging 2 and 5 days after 5-AzaC addition. Since cells were at a high confluence at day 2, the morphology changes over time could not be easily appreciated. However, a decrease in cell number was observed, this being clearer at 5 days for both concentrations added in multiple occasions (**Figure SM 2**).

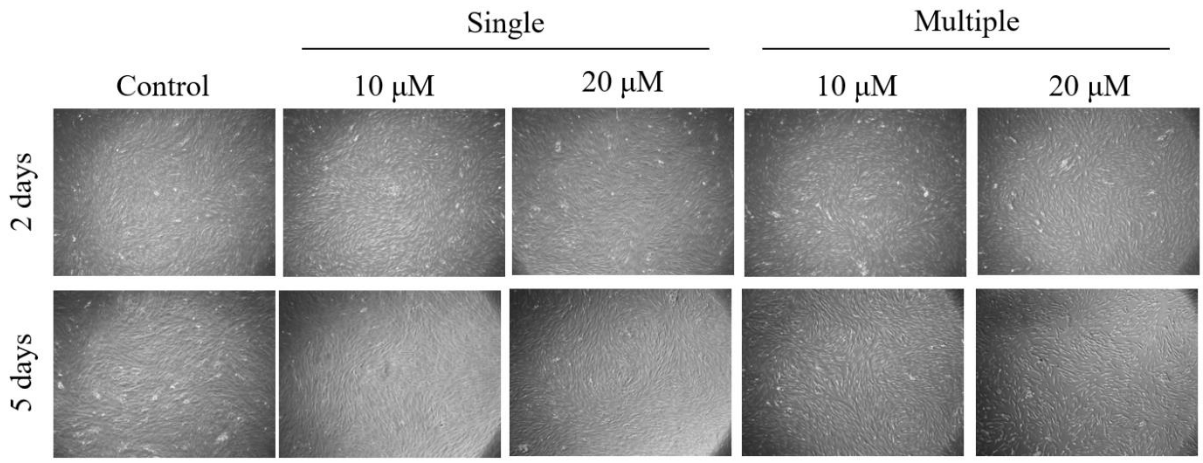

Figure SM 2. Images of hMSCs cultured for 2 and 5 days with 10 and 20 µM 5-AzaC that was either added in a single or in multiple additions. Images of cells cultured with plain medium are included as comparison.

Effect of gelatin coating on the chips

Since the chip channel was coated with gelatin to improve cell attachment, the effect of gelatin on the genetic expression of hMSCs was studied. For this purpose, gelatin-coated well plates were prepared. 0.2% type A gelatin (Sigma G9136) was dissolved in distilled water and autoclaved. 1 ml of gelatin solution was added into the wells, incubated at 37°C for 30 min and rinsed once with PBS before cell seeding. hMSCs were cultured in well plates with 5-AzaC for 5 days.

The results did not show any differences on the upregulation of genes independently of whether the wells had been previously coated with gelatin or not. This applied for both culturing conditions in which a single addition (**Figure SM 3**) and multiple additions of 5-AzaC were performed (data not shown).

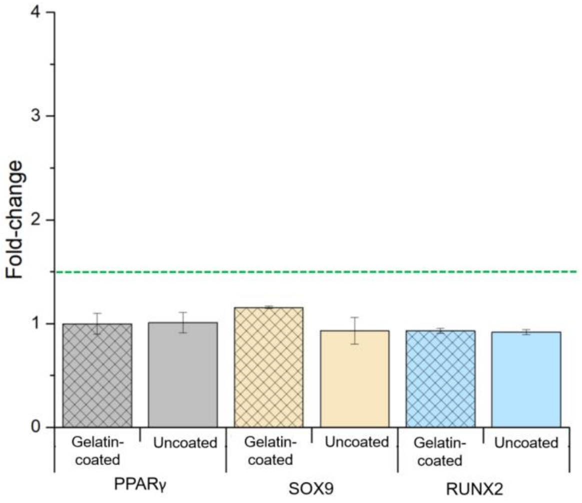

Figure SM 3. qPCR assessment of PPARγ, SOX9 and RUNX2 genes for hMSCs cultured for 6 days either in gelatin-coated wells or uncoated wells. The cell cultures were supplemented with 20 μM of 5-AzaC in a single addition for 5 days. The upregulation level (fold change > 1.5) is marked with a dash line. No statistically significant differences ($p < 0.05$) between samples within each master gene were found.

Effect of flow in the chips

The intrinsic shear-stress originated in a channel under flow could potentially modify the gene expression of hMSCs. To guarantee that the upregulation in the hMSCs was due to addition of the 5-AzaC and not the shear stress, a control experiment including flow but no addition of 5-AzaC was performed. None of the genes tested showed upregulation (fold-change < 1.5) due to flow in the microfluidic chip designed (**Figure SM 4**). Nevertheless, RUNX2, with a fold-change of 1.1 ± 0.1 showed a statistically significant higher fold-change than both PPARγ ($p < 0.001$) and SOX9 ($p < 0.001$).

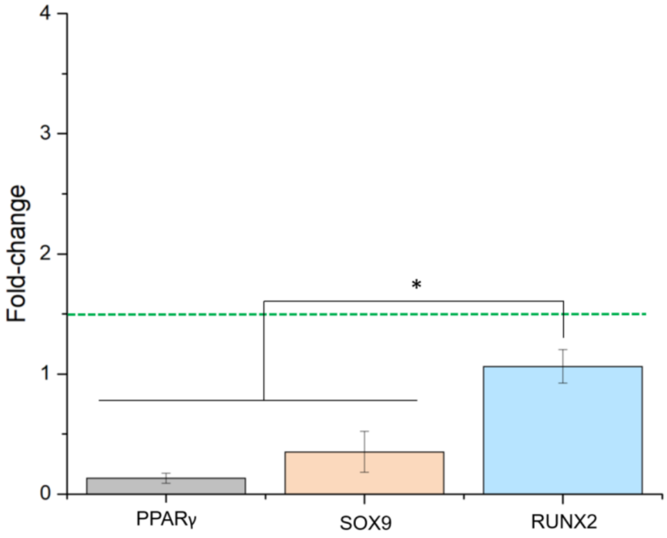

Figure SM 4. qPCR assessment of PPARγ, SOX9 and RUNX2 genes for hMSCs cultured on-chip with plain medium for 6 days to determine the effect of flow. The upregulation level (fold change > 1.5) is marked with a dash line. * indicates statistically significant differences ($p < 0.05$) between the master genes.

Statistics of the cytotoxicity assay

Table SM 1. P-values of the cytotoxicity results, where statistical differences in cell viability between each sample and the control were evaluated (significance level of $\alpha = 0.05$).

Sample types	p-value	
	2 days	5 days
Single addition of 10 μM 5-AzaC	0.393	0.975
Single addition of 20 μM 5-AzaC	0.025	0.015
Multiple addition of 10 μM 5-AzaC	0.077	0.000
Multiple addition of 20 μM 5-AzaC	0.007	0.000
